# Supplementary material for: Chassis-based fiber-coupled optical probe design for reproducible quantitative diffuse optical spectroscopy measurements
Source: PLoS One. 2024 Jul 25;19(7):e0305254. doi: 10.1371/journal.pone.0305254 (PMC11271963; doi:10.1371/journal.pone.0305254)
Supplement: Attachment — Submitted filename: Response To Reviewers.docx [file pone.0305254.s002.docx]

# Response to Reviewers

## Reviewer #1

**Reviewer 1, Comment 1**: Unfortunately, many figures in the manuscript have a very poor resolution (specifically the ones showing chassis construction). The manuscript would benefit by incorporating clear high resolution images of the chassis-based probe since it will be for sure interesting for other people working in the field.

Response: Thank you for bringing this to our attention. We have revised all figures to improve image resolution and clarity.

Changes to text: Figures 1-8 have been updated and formatted according to journal guideline.

## Reviewer #2:

**Reviewer 2, Comment 1**: It is surprising that using the chassis-mode the reproducibility is similar to the manual one and it decreases only for THC. The authors highlighted that when the probe was manually positioned, a marker was facilitating the procedure, reducing the CV. However, can you comment: why you do not see this improvement only on THC? Moreover, compared to the cited paper I was expecting an enhancement in the CV.

Response: Thank you for your comment. As you astutely noted, we did have modest improvement in THC in the chassis versus manual measurements but not in other parameters (StO2 or BFI). We believe the general comparability between the two methods is a reflection of “best case” attachment and reflects well on the chassis-based measurement because the manual method was precisely aligned each time.

Regarding the comparison of our results to prior work, our COV in BFI was ~19% and is a moderate improvement compared to prior reports of 27% in Andresen et al. We will make note of this in the discussion. The comparable COV of StO2 may reflect limitations imposed by the SNR of the StO2 measurements itself. This is influenced by the signal-to-noise ratio (SNR) of the amplitude and phase measurements of the FD-DOS measurement which is impacted by detected light intensity levels and ambient light leakage. In the future, hardware advancements which reduce measurement and quantization noise could further reduce measurement variability.

Changes to text: In “Discussion”, we added text to reflect that our BFI COV was moderately improved compared to prior work. Further we added additional discussion regarding the potential role of SNR in measurement variability and opportunities for future improvement with improved instrumentation.

**Reviewer 2, Comment 2**: It is not clear to me if the probe make use of optical prisms, or mirror to deflect the light of 90°? Are the fibers perpendicular or parallel to the tissue?

Response: Thank you for your astute question. Yes, we used optical prisms and 90-degree bend fibers to deflect light at a 90-degree angle.

Changes to text: In Methods, we clarified the use of prism-coupled optical fiber bundles and 90-degree bend optical fiber bundles.

**Reviewer 2, Comment 3**: In the abstract, line 55: “Of the eight measurements, four were acquired with by placing the probe into a secured chassis, and four were visually aligned and manually held.”, please remove “with”

Response: Thank you for catching this typo. We have corrected the text.

Changes to text: “with” removed in the abstract.
